# Supplementary material for: Antiviral Effect of Manganese against Foot-and-Mouth Disease Virus Both in PK15 Cells and Mice
Source: Viruses. 2023 Jan 30;15(2):390. doi: 10.3390/v15020390 (PMC9964130; doi:10.3390/v15020390)
Supplement: Supplementary file 1 [file viruses-15-00390-s001.zip › viruses-2112479-supplementary.pdf]

Table S1. Primers and probes used in this study.

Gene	Primer/Probe	Sequence 5′ to 3′
FMDV	Forward primer	ACTGGGTTTTAYAAACCTGTGATG
	Reverse primer	TCAACTTCTCCTGKATGGTCCCA
	Probe (6'FAM/MGB)	ATCCTCTCCTTTGCACGC
IFN-α	Forward primer	TCTGCAAGGTTCCCAATGG
	Reverse primer	AGATGGCATTGCAGCTGAGTAG
IFN-β	Forward primer	GCTAACAAGTGCATCCTCCAAA
	Reverse primer	AGCACATCATAGCTCATGGAAAGA
Mx	Forward primer	GAGGTGGACCCCGAAGGA
	Reverse primer	CACCAGATCCGGCTTCGT
OAS	Forward primer	AAGCATCAGAAGCTTTGCATCTT
	Reverse primer	CAGGCCTGGGTTTCTTGAGTT
ISG54	Forward primer	CTGGCAAAGAGCCCTAAGGA
	Reverse primer	CTCAGAGGGTCAATGGAATTCC
CCL5	Forward primer	TACAGCTACCATGAAGGTCTCCAC
	Reverse primer	TGATGCTGGAGCGCAGAG
GAPDH	Forward primer	ACATGGCCTCCAAGGAGTAAGA
	Reverse primer	GATCGAGTTGGGGCTGTGACT
